# Academic detailing to increase colorectal cancer screening by primary care practices in Appalachian Pennsylvania

**DOI:** 10.1186/1472-6963-11-112

**Published:** 2011-05-23

**Authors:** William J Curry, Eugene J Lengerich, Brenda C Kluhsman, Marie A Graybill, Jason Z Liao, Eric W Schaefer, Angela M Spleen, Mark B Dignan

**Affiliations:** 1 Department of Family and Community Medicine, College of Medicine, The Pennsylvania State University, Hershey, PA 17033-0850, USA; 2 Penn State Ambulatory Research Network, The Pennsylvania State University, Hershey, PA 17033-0850, USA; 3 Department of Public Health Sciences, College of Medicine, The Pennsylvania State University, Hershey, PA 17033-0855, USA; 4 Penn State Hershey Cancer Institute, The Pennsylvania State University, Hershey, PA 17033-0850, USA; 5 Department of Internal Medicine, University of Kentucky, Lexington, KY 40506-0093, USA

## Abstract

**Background:**

In the United States, colorectal cancer (CRC) is the third most frequently diagnosed cancer and second leading cause of cancer death. Screening is a primary method to prevent CRC, yet screening remains low in the U.S. and particularly in Appalachian Pennsylvania, a largely rural area with high rates of poverty, limited health care access, and increased CRC incidence and mortality rates. Receiving a physician recommendation for CRC screening is a primary predictor for patient adherence with screening guidelines. One strategy to disseminate practice-oriented interventions is academic detailing (AD), a method that transfers knowledge or methods to physicians, nurses or office staff through the visit(s) of a trained educator. The objective of this study was to determine acceptability and feasibility of AD among primary care practices in rural Appalachian Pennsylvania to increase CRC screening.

**Methods:**

A multi-site, practice-based, intervention study with pre- and 6-month post-intervention review of randomly selected medical records, pre- and post-intervention surveys, as well as a post-intervention key informant interview was conducted. The primary outcome was the proportion of patients current with CRC screening recommendations and having received a CRC screening within the past year. Four practices received three separate AD visits to review four different learning modules.

**Results:**

We reviewed 323 records pre-intervention and 301 post-intervention. The prevalence of being current with screening recommendation was 56% in the pre-intervention, and 60% in the post-intervention (p = 0. 29), while the prevalence of having been screened in the past year increased from 17% to 35% (p < 0.001). Colonoscopies were the most frequently performed screening test. Provider knowledge was improved and AD was reported to be an acceptable intervention for CRC performance improvement by the practices.

**Conclusions:**

AD appears to be acceptable and feasible for primary care providers in rural Appalachia. A ceiling effect for CRC screening may have been a factor in no change in overall screening rates. While the study was not designed to test the efficacy of AD on CRC screening rates, our evidence suggests that AD is acceptable and may be efficacious in increasing recent CRC screening rates in Appalachian practices which could be tested through a randomized controlled study.

## Background

In the United States (U.S.), colorectal cancer (CRC) is the third most frequently diagnosed cancer and the second leading cause of cancer death [1]. The American Cancer Society (ACS) estimates that in 2010, more than 140,000 new cases of CRC will be diagnosed and over 50,000 deaths will be attributed to CRC [1]. Screening is considered one of the best methods to prevent CRC [2]; the Centers for Disease Control and Prevention (CDC) estimates that 50-60% of CRC deaths could be prevented if all individuals aged 50 years and older had regular screening tests [3]. The ACS recommends CRC screening of average-risk individuals (defined as having no personal or family history of CRC and no personal history of bowel disease) starting at age 50 years. Recommended tests include: fecal occult blood test (FOBT)/fecal immunochemical test (FIT) annually, flexible sigmoidoscopy (FS) every 5 years, air contrast barium enema every 5 years, computerized tomography (CT) colonography every 5 years, or colonoscopy every 10 years [4].

Despite these recommendations, CRC screening remains low in the U.S., with only 61% of average-risk individuals being current with screening recommendations. In Appalachian Pennsylvania (PA), the CRC screening rate was less, at only 44.4% [5]. Spanning all or a portion of 13 states in the eastern U.S. including Pennsylvania, Appalachia is characterized by high rates of poverty, low health insurance coverage, and limited access to health care. In addition, CRC incidence and mortality rates have increased [6-8].

Receiving a physician recommendation for CRC screening is a primary predictor for patient adherence with screening guidelines [9,10]. Recognizing the pivotal role of the primary care provider, the Task Force on Community Preventive Services (Task Force) reviewed reports of provider-oriented interventions to increase screening for breast, cervical and colorectal cancer [11]. The Task Force found sufficient evidence to recommend two interventions: provider assessment and feedback, and provider reminders (inform provider that client screening is due) and recall (signifies client screening is overdue). Provider assessment and feedback interventions evaluate provider performance in delivering or offering screening to clients (assessment) and present providers with information about their performance in providing screening services (feedback) [10-14]. However, the studies upon which the recommendations related to CRC screening were based only upon FOBT as the screening modality and were relatively dated, having been published in 1986, 1989, and 1990 [15-17].

One interventional strategy to disseminate these evidence-based provider-oriented interventions is academic detailing (AD), a method that transfers knowledge or methods to physicians, nurses or office staff through the visit(s) of a trained educator. Academic detailers can provide methods to improve usage of the targeted service through assessment and feedback, and recalls and reminders. Indeed, physicians reported that AD is more likely to persuade them to implement CRC management guidelines than other strategies [18]. Acceptance of AD by primary care physicians has been generally high; 88% of Belgian physicians who had experience with AD indicated they would welcome similar visits in the future [19].

As a dissemination strategy, AD can be multi-component, which is also recommended by the Task Force, possibly including client-oriented interventions (e.g., client reminders, small media) and interventions to reduce structural barriers (e.g., inconvenient hours/locations, need for multiple practice visits) [14]. However, there are only a few reports of the efficacy of AD to increase cancer screening. In a 2x2 factorial randomized controlled trial in ten urban primary care practices, AD with patient reminders improved CRC screening with endoscopy by 14% [20]. In a second randomized controlled trial of underserved urban participants within eight community health centers, AD significantly improved CRC screening (any screening method) by 16% [21]. Another randomized controlled trial of urban populations served by 264 medical offices found a 7% increase in colonoscopy rates for the AD intervention arm [22]. A group randomized implementation trial in 32 primary care practices found a 4.9% (adjusted) increase in CRC screening (colonoscopy, FS, and FOBT) and 7.9% increase in CRC screening recommendation due to an AD with audit and feedback intervention. For breast cancer screening, a randomized block study of 168 primary care physicians found that AD intervention increased recommendation for mammography and practical breast examinations in an urban community [23]. Finally, a randomized controlled trial of 16 practices found that AD with chart audit and feedback was associated with a significant increase in recommendation for and receipt of breast cancer screening in Oklahoma [24].

While these previous studies suggest that an AD intervention to increase CRC screening in an urban setting is efficacious, there are no published reports of its efficacy among rural Appalachian populations. Unlike urban settings, rural areas have few primary care providers and residents often must travel long distances for a practice visit. The objective of the current study was to determine the acceptability and feasibility of an AD strategy among primary care practices in the rural, medically underserved Appalachian PA to increase CRC screening.

## Methods

A multi-site, practice-based, intervention study with pre- and 6-month post-intervention review of randomly selected medical records, pre- and post-intervention surveys, as well as a post-intervention focus group of study practices was conducted. The primary outcome was patients being current with CRC screening recommendations and having received a CRC screening within the past year. A secondary outcome was the presence of a CRC screening recommendation recorded in the medical chart. This research was conducted from February 2008 through January 2009. All materials and procedures were approved by the Penn State Milton S. Hershey Medical Center Institutional Review Board.

We recruited primary care practices (n = 4) in four counties of north-central PA through existing partnerships with ACTION Health Cancer Task Force, a community-based coalition of Northern Appalachia Cancer Network (NACN) and Penn State Ambulatory Care Research Network (PSARN). The NACN is a regional member of the National Cancer Institute (NCI)-funded Appalachia Community Cancer Network, whose goal is to develop, test and disseminate evidence-based interventions that measurably reduce cancer-related health disparities [7,8]. PSARN aims to link primary care research with patient care, medical education, and community service in central PA [25]. Three practices are members of a large multi-practice primary care health network which shares a full electronic medical record (EMR), but are geographically separated, while the fourth practice is affiliated with a major academic health system and uses a paper-based record.

We planned the study using the PRECEDE/PROCEED conceptual model for population health improvement, which guides users through a systematic process to identify factors that influence health and health behaviors [26-29]. The development of the study procedures included key informant interviews of medical office assistants, nursing staff, office administrators, and providers to assess current policies for preventive health care and screening practices at each participating practice. In addition to interviews, we conducted an environmental assessment of the practice, including screening tools, medical record flow sheets, computerized databases, patient education handouts, and patient brochures on CRC screening.

The health care providers, physicians and nurse practitioners, at each practice answered a health care provider survey. The survey instrument collected information on screening practices, follow-up and referral procedures, and characteristics of the practice. A lead nurse at each practice distributed the survey after the practice agreed to participate in the study and again after data collection was completed at each site. The health care provider survey sought to provide the research team with important information about the early detection of CRC, including screening recommendation behavior, opinions about screening, and some general questions about the individual medical practice. Once the survey was completed, the answers were transcribed and saved to a database. Unique identifiers for the respondents were established by the lead nurse of the clinic so surveys before and after the intervention could be linked. The list of identifiers was not shared with the research team providing anonymity for respondents.

Upon completion of the pre-intervention practice assessment, each of the four practices received three separate AD visits that reviewed four different learning modules. The intervention was delivered by a physician-led team including, a senior nurse and PhD-level behavioral health specialist who were trained by the lead physician. The academic detailers covered various topics (Table 1), reinforced standard medical practice, supplied providers and staff with county-specific cancer incidence and mortality data and ACS educational tools (e.g., wall poster, client reminders, scripts).

**Table 1:** Summary of information reviewed during academic detailing sessions

Learning Module	Title	Description of Academic Detailing Module
**Module A**	Screening Effectiveness/Guidelines	In-depth information about colorectal screening tests (benefits, barriers, costs, risks); current CRC screening guidelines.

**Module B**	Reimbursement Guidelines and Referral Services	Reimbursement guidelines for Medicaid, Medicare; Information about out-of-pocket expenses for colorectal cancer screening tests; Information about local referral sources available for colorectal cancer screening.

**Module C**	Patient Counseling	Insight and tools for discussion with apprehensive patients about colorectal cancer screening.

**Module D**	Screening and Follow-up	Tools to alert patients and providers when screening is due and tools to follow-up with patients to ensure they receive recommended screening.

Outcome measures were collected through medical chart abstraction performed prior to the AD intervention and six-months after the intervention. Abstraction was completed by abstractors hired from the participating practices. Abstracted data were entered directly into NCI's BioInformatics Grid^® ^(caBIG) through Oracle C3D. Developed to support translational and clinical research in oncology, the caBIG is a nationwide computer-based network "grid" of data-sharing tools and medical data intended to improve cancer care [30]. The abstraction protocol guided the abstractor through each step, including selection of medical records, determination of eligibility, recording screening results, and follow-up care.

Eligibility for medical record abstraction included patients seen in the practice during the previous two months who were aged 50 years or older. Ineligibility was conferred to patients seen for acute reasons, those with symptoms of CRC (e.g., rectal bleeding), and those with a prior diagnosis of CRC. The primary outcome measures were being current with CRC screening recommendation using FOBT, FS, or colonoscopy and having received a CRC screening of FOBT, FS, or colonoscopy during the previous year. We also documented the presence of a recommendation of CRC screening during the previous year as a secondary outcome measure. Information abstracted from the chart included age group in increments of 5 years, family history of CRC, interval since the previous physician visit, and any completed CRC screening tests along with their results. Patient names or other identifiers were not recorded in caBIG.

The prevalence of the outcome measures were calculated by dividing the number of patients who had a CRC screening (or recommendation) by the total number of eligible medical records reviewed. The statistical significance of differences in the estimates of the pre- and post-intervention screening rates were compared using chi square, or Fishers exact tests if the expected cell size was small. We used Wilcoxon tests to examine differences in median time to colonoscopy follow-up.

To assess acceptability and participant experiences in the intervention, a qualitative evaluation with key informant interviews was used to examine intervention processes, including participant perceptions of the program and consistency with programmatic goals. This was accomplished at the completion of chart data collection.

Semi-structured in-depth, face-to-face interviews with physicians, other health care providers and medical office directors were conducted by a trained qualitative researcher who was not a member of the research team, to insure open dialogue with interviewees. The semi-structured interview format sought to elicit specific information about the content and conduct of the intervention, including the acceptability of the intervention to providers and office staff, perceptions of what did and did not work as desired, and ways in which the provision of academic detailing could be improved.

The interview was tape recorded and transcribed. All names and other identifying information were removed from the transcript before the research team was able to review for content analysis.

## Results

### Medical Charts

We randomly selected 333 records for the pre-intervention analysis and 350 for the post-intervention. Of these, we eliminated 59 (8.6%) records because: 35 were selected in both the pre- and post-intervention review; 19 indicated a personal history of CRC or CRC symptoms; 4 did not include data on the type of CRC screening test; and 1 did not include screening data. Our final data set included 323 pre-intervention and 301 post-intervention patients. At pre-intervention, we observed no difference in age between the four practice sites; however a significant difference was present post-intervention with the academic practice having more patients in the 50-54 age range.

Overall, 182 (56%) of pre-intervention patients were current with CRC screening recommendations while 182 (60%) of post-intervention patients were current (p = 0.29). One practice had a significant increase in being current with recommendations, from 10 (21%) at pre-intervention to 20 (53%) post-intervention (p < 0.01). Additionally, 56 (17%) of pre-intervention patients had completed CRC screening in the year prior to the intervention compared to 106 (35%) of post-intervention patients (p < .001) (Table 2).

**Table 2:** CRC screening and recommendation per site

	Practice site	Pre-Intervention	Post-Intervention	p-value
**Current with**	1	**46**, 23, (50)	**41**, 19, (46)	0.73
**screening**	2	**50**, 39, (78)	**49**, 40, (82)	0.65
**Charts reviewed**,	3	**48**, 10, (21)	**38**, 20, (53)	**<0.01**
number current	4	**179**, 110, (61)	**173**, 103, (60)	0.71
(% current)	Overall	**323**, 182, (56)	**301**, 182, (60)	0.29

**Screened in past year**	1	**46**, 12, (27)	**41**, 21, (51)	**0.02**
**Charts reviewed**,	2	**50**, 6, (12)	**49**, 39, (80)	**<0.01**
number	3	**48**, 5, (10)	**38**, 22, (58)	**<0.01**
screened	4	**179**, 33, (18)	**173**, 24, (14)	0.25
(% screened)	Overall	**323**, 56, (17)	**301**, 106, (35)	**<0.01**

**Recommended, past year**	1	**46**, 34, (74)	**41**, 22, (54)	**0.05**
**Charts reviewed**,	2	**50**, 30, (60)	**49**, 10, (20)	**<0.01**
number recommended	3	**48**, 31, (65)	**38**, 24, (63)	0.89
(% recommend)	4	**179**, 43, (24)	**173**, 24, (14)	**0.02**
	Overall	**323**, 138, (43)	**301**, 80, (27)	**<0.01**

**Current in Screening **	1	**46**, 43, (94)	**41**, 35, (85)	0.21
**Charts reviewed**,	2	**50**, 46, (92)	**49**, 43, (88)	0.48
number accomplished	3	**48**, 34, (71)	**38**, 37, (97)	**<0.01**
recommended past year	4	**179**, 131, (73)	**173**, 117, (68)	0.25
(% accomplished)	Overall	**323**, 254, (79)	**301**, 232, (77)	0.64

Of the 364 patients who had been screened, colonoscopy was performed for 279 (76.7%), home FOBT for 79 (21.6%), and FS for 6 (1.7%). Of the CRC screening tests, 145 (39.8%) were recorded as normal, 143 (39.3%) as abnormal, and 76 (20.9%) not recorded. Of the not recorded, 47 (67.1%) were among post-intervention patients. The median time from date of colonoscopy recommendation to date of colonoscopy completion was 2 months. The pre-intervention group had a range of 0 to 11 months, and the post-intervention group had a range of 0 to 8 months (p = 0.82).

A recommendation for CRC screening was documented in 138 (43%) charts at pre-intervention, while 80 (27%) recommendations were documented at post-intervention (p < 0.01). Recommendations included FOBT, FS and colonoscopy. Despite not having a recommendation for a screening, 24 patients post-intervention were screened in the year. Furthermore, 86 patients who had no recommendation for screening in the past year had evidence they were current with screening. Of the 86 patients, 15 (17%) were among pre-intervention patients and 71 (83%) were post-intervention patients.

Paper-based charts comprised 179 of the pre-intervention reviews and 173 charts in the post-intervention review, while EMR charts comprised 144 and 128 of the reviews respectively. Practices with EMRs showed significantly improved documentation of CRC screening, from 72 (50%) to 76 (62%) (p = 0.05) while the practice using paper records had no change [111 (62%) pre-intervention; 104 (60%) post-intervention]. Screening prevalence in the past year increased in the EMR practices [23 (16%) to 82 (64%), p < 0.01] but not in the paper-based practice [32 (18%) to 24 (14%), p = 0.25]. When current recommendation and completed CRC screening were combined, the EMR practices' had significantly more patients receiving either in both the pre-intervention [122 (85%) vs. 131 (73%), p = 0.01] and the post-intervention [115 (90%) vs. 116 (67%), p < 0.01] (Table 3). The majority of undocumented results were from the EMR practices with 22 (31%) pre-intervention records and 42 (53%) post-intervention records missing the source documents. Less than 4% of screening results were missing in the paper medical records.

**Table 3:** CRC screening and recommendation by type of medical record

	Medical Record Type	Pre-Intervention	Post-Intervention	p-value
**Current with screening** **Charts reviewed**,	EMR	**144**, 72, (50)	**128**, 79, (62)	0.05
number current (% current)	Paper	**179**, 111, (62) p = 0.04	**173**, 104, (60) p = 0.70	0.71

**Screened in past year** **Charts reviewed**,	EMR	**144**, 23, (16)	**128**, 82, (64)	<0.01
number screened (% screened)	Paper	**179**, 32, (18) p = 0.56	**173**, 24, (14) p < 0.01	0.25

**Recommended, past year** **Charts reviewed**,	EMR	**144**, 95, (66)	**128**, 56, (44)	<0.01
number recommended (% recommend)	Paper	**179**, 43, (24) p < 0.01	**173**, 24, (14) p < 0.01	0.02

**Screened or recommended past year** **Charts reviewed**,	EMR	**144**, 122, (85)	**128**, 115, (90)	0.27
number accomplished (% accomplished)	Paper	**179**, 131, (73) p = 0.01	**173**, 116, (67) p < 0.01	0.25

### Physician Survey

Surveys were returned from 15 (100%) providers before the intervention and 15 (100%) at the end of the study. Two providers left their practices in the six months between surveys, and two new providers joined practices in this time frame. Three of the providers who completed surveys were nurse practitioners. One-third of respondents stated they were full or part owner of the practice, one-third stated they were employees of a physician-owned practice, and the other one-third were employees of a hospital, clinic or university practice. All respondents worked in single specialty clinics. One half of the respondents were affiliated with a medical school as faculty - full-time, adjunct or clinical.

In the initial survey, four physicians noted they were 50 years of age or older, and all had been screened by colonoscopy. In the final survey, six providers noted they were 50 years or older. Five had been screened, four with colonoscopy and one with FOBT cards and flexible sigmoidoscopy.

Most providers felt that all methods of screening were somewhat effective, except colonoscopy which was rated as very effective. Eight respondents (53%) did not know about FIT prior to the intervention, all knew about it after when they listed it as either somewhat or very effective.

Prior to the intervention, all respondents stated that colonoscopy was most often recommended for screening; after the intervention, two respondents listed FOBT or FIT as most commonly used.

The providers had a realistic assessment of their current screening rates stating that 51-75% of their patients were current on both surveys. Before the Lunch and Learn sessions, there was a lack of knowledge about Medicare reimbursement for FIT, flexible sigmoidoscopy, and contrast barium enema. After, respondents still lacked knowledge about reimbursement for CRC screening tests except FIT, sigmoidoscopy and colonoscopy (Table 4).

**Table 4:** Provider Survey Summary

	Pre-Intervention			Post-Intervention		
Effectiveness of Screening (mode)						
FOBT	somewhat effective			somewhat effective		
FIT	don't know			somewhat effective		
FS	somewhat effective			somewhat effective		
Digital Rectal Exam	somewhat effective			somewhat effective		
Colonoscopy	very effective			very effective		
Double Contrast Barium Enema	somewhat effective			somewhat effective		

Approximately what proportions of your patients over age 50 are current with CRC screening? (Mode)	51-75%			25-50%		

To the best of your knowledge, does Medicare reimburse CRC screening in asymptomatic, average risk patients 65 and older?	No	Yes	Don't know	No	Yes	Don't know
FOBT	0	13	2	0	13	0
FIT	0	2	11	3	5	6
FS	4	1	9	3	4	6
Colonoscopy	1	13	1	0	13	0
Double Contrast Barium Enema	3	1	9	1	3	10

Four providers (27%) had received training in performing colonoscopy, and all respondents strongly agreed that this was an effective screening when performed by a well-trained primary care physician. Most agreed that colonoscopy was best performed in an endoscopy center. No respondents were currently performing colonoscopy, but three of the offices referred patients for screening colonoscopy to a family physician colonoscopist who is part of their practice network.

Mechanisms to identify patients in need of CRC screening were felt to be in place by 87% of respondents initially and by 100% after. Yet, less than 30% reported a mechanism to ensure that patients who were given FOBT/FIT kits actually completed and returned the kits.

### Post-study Key Informant Interviews

A physician, two nurses, two medical assistants and two administrators from the practices participated in the key informant interviews. Each practice site was represented.

Themes that emerged from the semi-structured interview included practice self-assessment; study design including who should attend AD sessions, and who should lead AD sessions; CRC screening test options for patients; and helpful resources for practices to promote CRC screening.

Each site's representative felt that their programs were mature in the processes of identifying those in need and providing screening opportunities.

Interviewees felt study design was adequate. AD was embraced as a reasonable option to spread information, but for information that was 'not novel', three sessions were too many for 'mature practices'. If new material or a novel intervention was presented, multiple sessions would be appropriate. The ability to present the short information details multiple times during each AD session, as providers could participate, was seen as a strength of this approach.

The detailing sessions were intended for all members of the practice yet it was felt that sessions be offered for clinicians separate from nursing and medical office assistant staff. When asked if a non-physician could present information similar to that presented, the group was in agreement that peer-to-peer detailing was preferred. Physicians should lead AD for the clinicians; a nurse educator would have been acceptable for non-provider staff. Providing the detailing in person was preferred over other formats such as a webinar.

The provision of patient educational materials was noted as a potent stimulus to improve CRC screening. An adult cancer screening wall chart from the ACS was well received by staff and patients alike. Multiple anecdotes were shared about how these charts prompted patients to ask about becoming current with their cancer screening. Likewise, the ACS patient brochures on CRC screening were felt to be excellent resources, but second preference to the wall chart. The group suggested that a Frequently Asked Questions (FAQs) sheet be provided for nurses and medical office assistants to have as a prompt for questions that might be asked when engaging patients in a discussion about screening or when scheduling screening tests.

Three major themes emerged. First, the FIT should be aggressively used in practices. Most of the providers were not familiar with FIT at the beginning of the intervention, nor were they aware of the ACS guidance about its use in screening. Second, it was uniformly agreed that "all members of the practice should do their part" to promote CRC screening. Finally, they stated that they all had improved their ability to offer screening in the practices.

## Discussion

Our study found that AD was an acceptable and feasible intervention strategy in Appalachian PA. The four practices recruited for the project completed the study. Representatives from each practice reported that AD was a good mechanism to engage practices in CRC screening process improvement. One low performing practice at the beginning of the intervention showed significant improvement in screening rates after the intervention and the percentage of patients screened in the year immediately prior to the AD intervention increased significantly after the AD intervention, suggesting that AD had a positive impact upon CRC screening.

Our pilot work did not show a significant change in screening rates overall. Three reasons may be possible. First, study sites had a relatively high pre-intervention rate of CRC screening. Second, ceiling effects for cancer screening have been described. Mammography screening rates have been reported to have ceiling effects between 90-95%, and CRC screening rates near 60% [31]. Finally, our follow-up chart audits occurred just six months after the intervention, yet had a look-back period of 12 months. In our pilot work, only four practices were recruited, and small sample size may have contributed.

Despite increased number of screenings in the charts audited after the intervention, the percentage of charts with a recommendation for CRC screening decreased. We are uncertain of the reason for decreased recommendation, but it may be that providers omitted documentation for a recommendation because they proceeded to schedule screening tests for their patients [32]. As with any study, random error due to chart selection may also have caused this observation.

While this study was not designed to examine the impact of practice characteristics upon CRC screening, these characteristics are worthy of discussion. First, the impact of AD on CRC screening differed between EMR practices and the paper-based medical record practice. A significant increase (16% to 64%) in records that had documentation of CRC screening currency was observed in the EMR practices but was not observed in the paper record practice. Similarly, when combining screening or recommendation for screening, the practices using an EMR had significantly higher rates combined than the paper record practice. This differential increase may be attributed to AD motivated providers to act on the EMR health maintenance reminders more frequently than before the intervention [33]. Second, practice processes may affect CRC screening rates. For example, the practices using the EMR required a healthcare team member (usually a nurse) to annually review and document age-appropriate disease prevention in the EMR to keep reminders current at the point of care for each patient [34]. Third, patient characteristics may also be a factor in the screening rates. For example, patients at the paper record site were significantly younger cohort in the post-intervention chart review [32,35,36].

Importantly, nearly one-third of completed screening tests in the EMR practices did not have documented results in either pre- or post- intervention review, compared to less than 4% of completed tests being undocumented in the paper record practice. At the transition from paper records to an EMR, paper data is often scanned into a database that does not have searchable data elements which makes searching for information cumbersome. Data abstractors may have overlooked documentation due to the cumbersome nature of these databases. A specified time range may also be employed to convert paper data into digital format. If a colonoscopy was performed before the start date of the specified window, results would not be available within the EMR. Future studies will need to include augmented abstractor training to insure the most accurate chart reviews of archived data.

As with all studies, our study has limitations. First, our study did not include comparison sites to control for secular trends in CRC screening. Second, only four sites participated in our study. Third, our follow-up period was limited to six months, thus inhibiting conclusions regarding the continued impact of the intervention. Finally, we were not able to examine the effect of the individual components of our AD intervention.

The study also has important strengths. First, the study was the first to examine AD to increase CRC screening in a rural population. Second, we developed methods that can be used in future studies. Importantly, we used the caBIG Oracle C3D web data collection tool which facilitated data entry within the practice sites and provided the research team with a data set for analysis.

The common data elements in caBIG will allow the NCI to aggregate information across a variety of research settings [37,38]. Finally, the study identified important areas for future research, including practice characteristics, such as the effects of medical recording systems, and patient characteristics.

Translation of this work to future studies and practice improvement should include education about the FIT, include fewer AD sessions and particularly be focused on practices which have lower CRC screening rates.

## Conclusions

AD is an acceptable and feasible method for primary care providers in rural Appalachia. While not designed to test the efficacy of AD on CRC screening rates, we found evidence to suggest that AD may be efficacious in increasing screening rates in some practices. Our results suggest that a randomized study of the impact of AD on CRC screening in rural Appalachia is feasible and may support AD as an evidenced-based method to increase CRC screening.

## List of Abbreviations

(AD): Academic Detailing; (ACS): American Cancer Society; (caBIG): Cancer Biomedical Informatics Grid; (CDC): Center for Disease Control and Prevention; (CRC): Colorectal Cancer; (CT): Computerized Tomography; (EMR): Electronic Medical Record; (FIT): Fecal Immunochemical Test; (FOBT): Fecal Occult Blood Test; (FS): Flexible Sigmoidoscopy; (NCI): National Cancer Institute; (NACN): Northern Appalachia Cancer Network; (PSARN): Penn State Ambulatory Research Network.

## Competing interests

The authors declare that they have no competing interests.

## Authors' contributions

WJC designed the study, conducted the AD, and led the writing of the manuscript, with the assistance of EJL. BCK and MAG participated in delivery of the AD and the writing of the manuscript. JZL and EWS conducted the statistical analysis. AMS contributed to the writing of the manuscript. MBD provided overall direction to the study and contributed to the writing of the manuscript. All authors read and approved the final manuscript.

## Pre-publication history

The pre-publication history for this paper can be accessed here:

http://www.biomedcentral.com/1472-6963/11/112/prepub
